# Prevalence and predictive importance of anemia in Swedish nursing home residents – a longitudinal study

**DOI:** 10.1186/s12877-016-0375-2

**Published:** 2016-12-02

**Authors:** Björn Westerlind, Carl Johan Östgren, Sigvard Mölstad, Patrik Midlöv

**Affiliations:** 1 Department of Geriatrics, County Hospital Ryhov, Region Jönköping County, Jönköping, Sweden; 2 Department of Medical and Health Sciences, Linköping University, Linköping, Sweden; 3 Department of Clinical Sciences in Malmö, Center for Primary Health Care Research, Lund University, Malmö, Sweden

**Keywords:** Anemia, Mortality, Elderly, Nursing homes, Longitudinal study

## Abstract

**Background:**

Anemia is common in elderly people and especially in nursing home residents. Few studies have been performed on the consequences of anemia in a nursing home population. This study explored the prevalence of anemia in nursing homes in Sweden, including risk factors and mortality associated with anemia or hemoglobin (Hb) decline.

**Methods:**

Three hundred ninety patients from 12 nursing homes were included during 2008–2011. Information about medication, blood samples, questionnaire responses and information about physical and social activities was recorded. The baseline characteristics of the patients were compared for subjects with and without anemia. Vital status was ascertained during the following 7 years from baseline to compare the survival. Hb levels <120 g/L in women and <130 g/L in men were used to define anemia. For 220 of the subjects Hb change during one year was registered and the quartiles in Hb change were compared in terms of baseline characteristics and mortality.

**Results:**

The prevalence of anemia at baseline was 52% among men and 32% among women. The men with anemia had a two-year mortality significantly higher (61%) than the men without anemia (29%, *p* = 0.001) but there was no statistical difference in two-year survival in women. In anemic men there was a higher mortality (Hazard Ratio = 1.58) during a total follow-up period of up to 7 years after adjustment for age, increased B-type natriuretic peptide (BNP) and decreased estimated Glomerular Filtration Rate (eGFR). Among men, but not women, we found baseline correlations between anemia and elevated BNP (>100 ng/L) and severely reduced eGFR (<30 ml/min). When the lowest quartile of Hb change (decline > 9 g/L) was compared with the highest (improvement > 6 g/L) the mortality was higher in the lowest quartile (*p* = 0.03).

**Conclusions:**

Anemia is common in nursing home residents in Sweden, especially among men for whom it is related to higher mortality. A rapid Hb drop is associated with higher mortality. Regardless of earlier Hb values, monitoring Hb regularly in a nursing home population seems important for catching rapid Hb decline correlated with higher mortality.

## Background

Anemia is a common medical condition which is considered a global health problem as it affects both developed and developing countries and all ages [1, 2]. Anemia is more common among older persons and the prevalence increases with advancing age [3–6]. Anemia in older adults is frequently associated with negative outcomes, including decreased physical performance, increased number of falls, increased frailty, increased hospitalization, increased cognitive impairment and increased mortality [7].

Anemia is defined by the World Health Organization (WHO) as hemoglobin (Hb) < 120 g/L in women and < 130 g/L in men [8]. These limits are established worldwide and used in the vast majority of publications on this topic [5]. The WHO limits have also been questioned, however, due to few stated references, the small number of reference subjects, and some methodological problems [9]. The relevance of the WHO limits in older adults has also been discussed as the reference sample was aged < 65 years [10]. Lower anemia limits for elderly patients in general have been suggested [11] as well as equal limits (i.e. lower limits for older men) for both sexes among the oldest old [12] and higher decision limits among older adults [13]. Older men have a tendency to have a slightly higher prevalence of anemia than women, which has been suggested to be a result of the sex specific limits [14]. Lower Hb limits in premenopausal women seem motivated due to menstrual blood losses, but could consequently be questioned in older women [13]. On the other hand, higher levels of androgens in men stimulate the hematopoietic system by various mechanisms and is correlated with the Hb level [15]. Still, despite being debated, the WHO limits are found appropriate and clinically relevant by several authors for older persons as well [5, 10, 16].

In a large community-based US population study (the Third National Health and Nutrition Examination Survey, NHANES III) 11.0% of the men and 10.2% of women aged 65 years and older were anemic. Among those older than 85 years 26% of men and 20% of women were anemic [14]. In NHANES III there are also considerable differences in anemia prevalence due to ethnicity [13]. The Health Survey for England (HSE) found a similar anemia prevalence among people over 65 years [10].

In older nursing home residents the reported prevalence of anemia is higher, between 48 and 67% [5, 17–22], compared to population-based studies. Among women in nursing homes the occurrence varies between 49 and 65% [17, 19, 20, 22, 23] and among men between 45 and 70% [17, 19, 20, 22].

Low levels of Hb are considered a marker of disease and associated with increased mortality [4, 16, 24]. Some studies indicate that low levels of Hb are a stronger risk factor for mortality in men than in women [10, 16, 25]. Even mild anemia is shown to be associated with a higher mortality rate in elderly persons [25].

The main causes of anemia in the elderly population are nutrient-deficiency-related anemias, anemia due to chronic disease or chronic inflammation and anemia due to chronic kidney disease (CKD), but there is also a high rate of unexplained anemia [4, 14]. In nursing home residents CKD has been highlighted as an important cause of anemia [18, 20, 21, 26].

It is widely held that there is a need for more research on how to interpret levels of Hb in the oldest old with frailty [4, 5, 27].

The aim of this study was to explore the prevalence of anemia in an elderly Swedish population living in nursing homes, with special consideration of risk factors and mortality associated with anemia or rapid Hb decline.

## Methods

### Study population

The Study of Health and Drugs in the Elderly (SHADES) was a longitudinal cohort study of elderly people living in 12 different nursing homes in three cities in the south of Sweden (Jönköping, Linköping, and Eslöv). The aim of the SHADES study was to describe and analyze the morbidity, mortality, laboratory findings and pharmaceutical treatment of elderly residents in nursing homes and to use the data collected to improve health care [28].

In the 12 selected nursing homes all residents were invited to join the study. When a resident living in one of the nursing homes moved or died, the next person who moved in to the nursing home was invited to participate. During 2008–2011, 428 patients were included in the SHADES study. Persons living in a nursing home only temporarily for palliative care or short-term rehabilitation were excluded, as well as individuals with language difficulties and persons under the age of 65. Written informed consent was obtained from all participants. For those participants who could not understand the information and give informed consent due to cognitive impairment this was obtained from next of kin.

### Methods of investigation

**Fig. 1:**
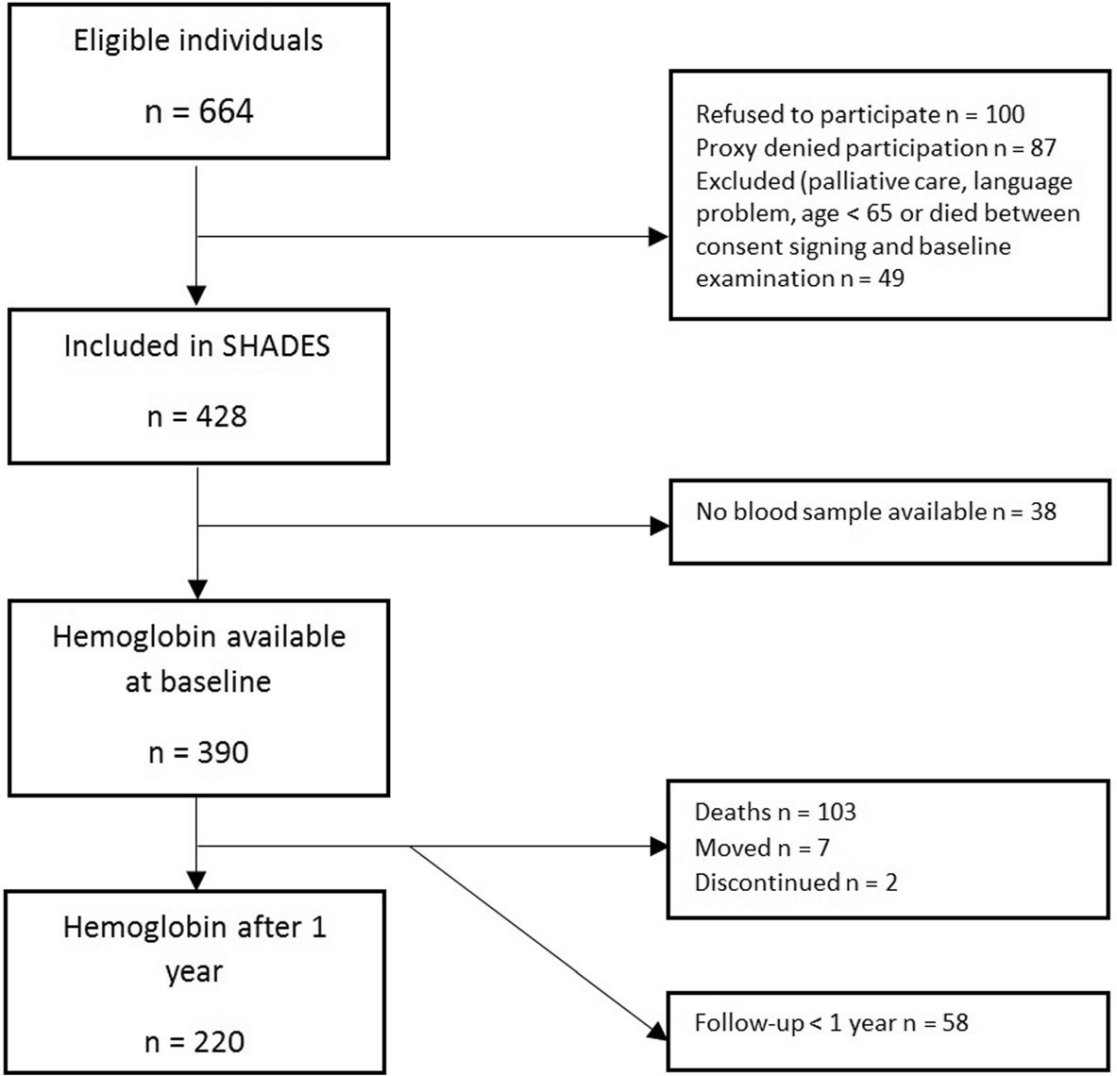
Flow chart of patients in the SHADES study

Participants were examined at baseline by specially trained nurses who also collected data from patient records for current medical treatment. The in-person testing of participants was performed by the study nurses with assistance of the staff at the nursing home and included measurement of weight and height. The Mini Mental State Examination (MMSE) [29] was used to measure cognitive function. MMSE assesses cognitive function through a number of questions directed to the participant regardless of cognitive function. Nutrition status was assessed through Short-form Mini-nutritional assessment (MNA-SF) [30]. Nutrition assessment with the MNA scale does not require cooperation from the participant, but information from the nursing home staff. Time weekly for physical activities and social activities for each individual was estimated by the staff of the nursing home. Blood samples were drawn according to a standard procedure. In 390 of the 428 included subjects baseline Hb analysis was performed. The analyses of Hb were performed in connection with the sampling in the local laboratories of the hospitals in Jönköping, Linköping and the Health Center in Eslöv. Reasons for a missing Hb analysis were lack of patient collaboration or blood sampling difficulties. The numbers of participants included, excluded, or missing in the different parts of study are illustrated in Fig. 1.

Remaining blood samples were stored at −70 °C in a freezer. Levels of creatinine, cystatin C, B-type natriuretic protein (BNP), transthyretin, C-reactive protein (CRP), ferritin, and transferrin were analyzed at the laboratory in the County Hospital Ryhov in Jönköping by high-pressure liquid chromatography. For BNP, transthyretin, CRP, ferritin, and transferrin we used the routine cut-off values suggested by the laboratory. For assessing renal function we used the formula for estimating glomerular filtration rate (GFR) according to recently updated Swedish guidelines [31]. The estimated GFR (eGFR) was defined as the average of (1) the GFR estimated from creatinine based on the revised equations for estimating GFR from the Lund-Malmö Study cohort [32] and (2) the GFR estimated from cystatin C with the CAPA formula [33].

We also followed the subjects during a follow-up time of up to 7 years after inclusion with death dates from the Swedish population register.

The population with 12-month follow-up values for Hb was divided into quartiles according to change in Hb value. The lowest quartile of Hb change (decline > 9 g/L) was compared to the highest (improvement > 6 g/L) in terms of baseline characteristics and mortality.

### Statistical analysis

All statistical analyses were performed using SPSS Statistics version 23 (SPSS, Inc. Chicago, IL). Since we assumed that the mean values were normally distributed we used Student’s *T*-test for continuous variables and we used the Chi-squared test for discrete variables. Survival analysis was performed using Cox regression (proportional hazards analysis) with a follow up time of up to about 7 years, the subjects still alive 1 January 2016 were considered as censored.

## Results

There were 276 women (71%) and 114 men (29%) in the study cohort. The mean age at inclusion was 85.1 years (±6.8) with a range between 65 and 101 years. The female subjects were older (85.9 ± 6.6 years) than the male subjects (83.0 ± 6.7, *p* < 0.001).

According to the WHO limits 52% of the men and 32% of the women had anemia (130 and 120 g/l respectively). When we used the same anemia limit for men as for the women (120 g/L) the anemia occurrence was considerably lower (27%), with no significant difference from women (*p* = 0.325). The women with anemia according to the WHO limits were significantly older than the non-anemic women. Men had the same tendency but the age difference was not significant.

There were no significant differences in weight, height, BMI, time for physical activity, or social activity between women with and without anemia. Among men, the only difference was that the men with anemia were less physically active. On average study subjects were taking 6.8 drugs daily and no statistically significant differences were observed between men and women or subjects with and without anemia. Subjects with anemia did not use drugs that could increase the bleeding risk more frequently than non-anemic subjects (Table 1).

**Table 1:** Baseline characteristics of the women and men with and without anemia

Women				
Parameter	All (*n* = 276)	Anemia (A) = Hb < 120 g/L (*n* = 89)	Nonanemic (NA) (*n* = 187)	*p*-value (A vs. NA)
	100%	32.2%	67.8%	
Age (years) mean ± SD	85.9 ± 6.6	87.1 ± 6.0	85.4 ± 6.8	0.037
Hb (g/L) mean ± SD	124.5 ± 13.6	109.0 ± 7.5	131.8 ± 8.7	<0.001
Weight (kg) mean ± SD	63.9 ± 14.0	62.0 ± 13.7	64.7 ± 14.1	0.137
Height (cm) mean ± SD	159.6 ± 6.8	159.0 ± 6.5	159.8 ± 6.9	0.370
BMI (kg/m^2^) mean ± SD	25.0 ± 5.0	24.6 ± 5.2	25.3 ± 5.0	0.296
Physical activity (h/week) mean ± SD	0.51 ± 0.76	0.43 ± 0.69	0.54 ± 0.79	0.246
Social activity (h/week) mean ± SD	1.97 ± 2.32	1.97 ± 2,28	1.97 ± 2,34	0.983
Medications in total ± SD	6.8 ± 3.0	7.0 ± 3,3	6.7 ± 2,9	0.442
Warfarin	7.2% (*n* = 20)	3.4% (*n* = 3)	9.1% (*n* = 17)	0.087
ASA	50.7% (*n* = 140)	46.1% (*n* = 41)	52.9% (*n* = 99)	0.286
SSRI	35.1% (*n* = 97)	39.3% (*n* = 35)	33.2% (*n* = 62)	0.316
NSAID	1.8% (*n* = 5)	1.1% (*n* = 1)	2.1% (*n* = 4)	0.554
BNP > 100 ng/L (*n* = 273)	50.5%	55.7%	48.1%	0.242
eGFR < 60 mL/min (*n* = 273)	61,9%	67.4%	59.2%	0.192
eGFR < 30 mL/min (*n* = 273)	7.0%	11.2%	4.9%	0.053
Transthyretin < 0.23 g/L (*n* = 272)	59.9%	68.5%	55.7%	0.043
CRP ≥ 10 mg/L (*n* = 275)	31.6%	46.6%	24.6%	<0.001
Ferritin > 204 μg/L (*n* = 276)	17.8%	25.8%	13.9%	0.015
Transferrin < 1.9 g/L (*n* = 275)	4.7%	6.8%	3.7%	0.262
Men				
Parameter	All (*n* = 114)	Anemia (A) = Hb < 130 g/l (*n* = 59)	Nonanemic (NA) (*n* = 55)	*p*-value (A vs NA)
	100%	51.8%	48.2%	
Age (years) mean ± SD	83.0 ± 6.7	84.2 ± 6.3	81.8 ± 7.0	0.064
Hb (g/L) mean ± SD	128.6 ± 15.4	117.1 ± 9.0	140.9 ± 10.6	<0.001
Weight (kg) mean ± SD	73.9 ± 13.1	71.9 ± 11.9	76.0 ± 14.0	0.095
Height (cm) mean ± SD	173 ± 6.7	172.4 ± 6.6	172.7 ± 6.9	0.811
BMI (kg/m^2^) mean ± SD	24.8 ± 4.3	24.2 ± 4.1	25.4 ± 4.3	0.129
Physical activity (h/week) mean ± SD (*n* = 109)	0.58 ± 0.94	0.39 ± 0.71	0.78 ± 1.11	0.030
Social activity (h/week) mean ± SD (*n* = 109)	1.19 ± 1.94	1.04 ± 1.98	1.35 ± 1.89	0.401
Medications in total	7.1 ± 3.2	7.6 ± 3,3	6.6 ± 3,0	0.078
Warfarin	11.3% (*n* = 13)	11.7% (*n* = 7)	10.9% (*n* = 6)	0.873
ASA	56.1% (*n* = 64)	52.5% (*n* = 31)	60.0% (*n* = 33)	0.423
SSRI	33.3% (*n* = 38)	30.5% (*n* = 18)	36.4% (*n* = 20)	0.508
NSAID	0.9% (*n* = 1)	1.7% (*n* = 1)	0.0% (*n* = 0)	0.332
BNP > 100 ng/L (*n* = 113)	49.6%	63.8%	34.5%	0.002
eGFR < 60 mL/min (*n* = 114)	61.4%	67.9%	54.5%	0.146
eGFR < 30 mL/min (*n* = 114)	11.4%	20.3%	1.8%	0.002
Transthyretin < 0.23 g/L (*n* = 114)	56.1%	62.7%	49.1%	0.143
CRP ≥ 10 mg/L (*n* = 112)	34.8%	45.8%	22.6%	0.010
Ferritin > 275 μg/L (*n* = 113)	15.0%	16.9%	13.0%	0.554
Transferrin < 1.9 g/L (*n* = 113)	3.5%	3.4%	3.7%	0.928

*Hb* hemoglobin, *SD* standard deviation, *BMI* Body Mass Index, *ASA* acetylsalicylic acid, *SSRI* selective serotonin re-uptake inhibitors, *NSAID* nonsteroidal anti-inflammatory drugs, *BNP* B-type natriuretic peptide, *eGFR* estimated glomerular filtration rate, *CRP* C-reactive protein

Among the male subjects with anemia the two-year mortality was significantly higher (61%) than among the subjects without anemia (29%, *p* = 0.001). Among female subjects we found no statistical difference in two-year survival (49% vs 43%, *p* = 0.340).

**Fig. 2:**
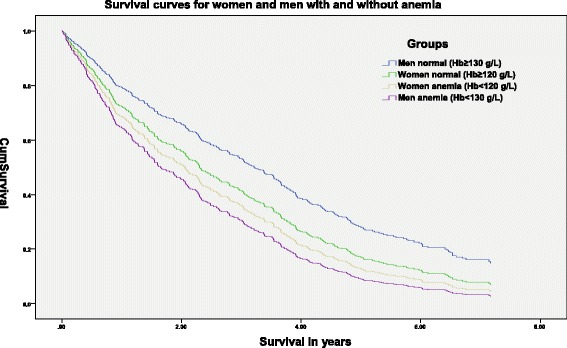
Survival curves for women and men with and without anemia

The difference in survival during the total follow-up time (up to about 7 years) is presented as survival curves for men and women with and without anemia (Fig. 2).

The difference in survival was not significant between anemic and non-anemic subjects in total or after adjustment for age, increased BNP and decreased eGFR.

However, when we stratified for sex, the difference in survival during the follow-up period among male subjects with or without anemia was significant (Hazard Ratio 1.58) even when adjusting for age, increased BNP and decreased eGFR (Table 2).

**Table 2:** Cox regression analysis including anemia, age, increased BNP level and decreased eGFR in relation to mortality

Variable	*p*-value	HR	CI 95% for HR
All subjects			
Anemia Hb < 120 g/L and 130 g/L	0.066	1.225	0.987–1.521
Age (years)	<0.001	1.051	1.033–1.070
BNP > 100 ng/L	<0.001	1.751	1.403–2.187
eGFR < 60 mL/min	0.863	0.980	0.782–1.228
Women			
Anemia Hb < 120 g/L	0.495	1.097	0.841–1.429
Age (years)	<0.001	1.043	1.022–1.066
BNP > 100 ng/L	<0.001	1.930	1.477–2.522
eGFR < 60 mL/min	0.286	0,866	0.666–1.128
Men			
Anemia (Hb < 130 g/L)	0.028	1.580	1.049–2.379
Age (years)	0.001	1.072	1.031–1.115
BNP > 100 ng/L	0.137	1.367	0.906–2.064
eGFR < 60 ml/min	0.403	1.215	0.770–1.919

*Hb* hemoglobin, *HR* hazard ratio, *CI* confidence interval, *BNP* B-type natriuretic peptide, *eGFR* estimated glomerular filtration rate

Among female subjects there was no significant correlation between anemia and mortality but we noted a correlation between increased BNP and mortality (Table 2).

When we investigated correlations between anemia and some potential causes of anemia, we also found differences between men and women (Table 1). Among men we found correlations between anemia and elevated BNP (>100 ng/L) and with severely reduced eGFR (<30 ml/min). These correlations were not seen among women. Among women we found correlations between anemia and several markers of inflammation, such as decreased transthyretin (<0.23 g/L), increased ferritin (>204 μg/L), and increased CRP (≥10 mg/L). Among men with anemia, there was a correlation with increased CRP, but not with increased ferritin (>275 μg/L) or transthyretin (<0.23 g/L, Table 1).

Prospective data after 12 months were used to compare the quartiles in Hb change. In the lowest quartile (decline more than 9 g/L) there was a higher mortality during the following 12 months (36%) compared with the other quartiles (18%, Table 3). The lowest quartile in Hb change had higher Hb at baseline compared with the highest quartile in Hb change (134 vs 113 g/L, *p* < 0.001), but we found no other significant differences including age, sex, number of drugs, Body Mass Index (BMI), eGFR, BNP, MNA-SF and MMSE (Table 3).

**Table 3:** Quartiles of change in Hb levels during 12 months and the subsequent 1-year mortality

Hb change range (g/L)	Quartile 1:	Quartile 2:	Quartile 3:	Quartile 4:	*p*-value
	−45 – –10 (*n* = 58)	−9 – −2 (*n* = 55)	−1 – +6 (*n* = 56)	+7 – +28 (*n* = 51)	(comparing q1 with q4)
Hb at baseline in men (g/L) mean ± SD	134.3 ± 11.0 (*n* = 22)	132.6 ± 9.9 (*n* = 16)	127.7 ± 14.0 (*n* = 15)	112.9 ± 11.3 (*n* = 12)	*p* < 0.001
Hb at baseline in women (g/L) mean ± SD	128.2 ± 10.8 (*n* = 36)	127.0 ± 13.0 (*n* = 39)	123.4 ± 10.9 (*n* = 41)	120.5 ± 13.1 (*n* = 39)	*p* = 0.007
Male sex (%)	37.9 (*n* = 22)	29.1 (*n* = 16)	26.8 (*n* = 15)	23.5 (*n* = 12)	*P* = 0.10
Age (years)	83.4 ± 8.3 (*n* = 58)	83.6 ± 6.3 (*n* = 55)	83.1 ± 7.3 (*n* = 56)	85.7 ± 5.8 (*n* = 51)	*p* = 0.10
Number of drugs, mean ± SD	7.2 ± 3.0 (*n* = 58)	6.7 ± 2.5 (*n* = 55)	6.8 ± 3.1 (*n* = 56)	6.7 ± 3.5 (*n* = 51)	*p* = 0.47
BMI (kg/m^2^) mean ± SD	25.5 (±5.1) (*n* = 58)	25.6 ± 5.0 (*n* = 55)	25.0 ± 4.4 (*n* = 56)	24.9 (±4.8) (*n* = 51)	*p* = 0.53
eGFR (mL/min)	56.8 ± 16.1 (*n* = 58)	55.7 ± 14.3 (*n* = 55)	51.9 ± 14.4 (*n* = 56)	54.2 ± 15.7 (*n* = 51)	*p* = 0.38
BNP (ng/L) mean ± SD	139.4 ± 190.2 (*n* = 57)	117.8 ± 166.4 (*n* = 54)	159.6 ± 235.8 (*n* = 55)	128.6 ± 114.0 (*n* = 51)	*p* = 0.72
MNA-SF initial result mean ± SD	10.5 ± 2.3 (*n* = 58)	10.9 ± 2.2 (*n* = 55)	10.8 ± 2.1 (*n* = 55)	10.4 ± 2.8 (*n* = 51)	*p* = 0.74
MMSE result mean ± SD	16.2 ± 6.7 (*n* = 49)	17.6 ± 6.0 (*n* = 50)	17.8 ± 6.5 (*n* = 49)	17.9 ± 6.2 (*n* = 49)	*p* = 0.20
1 year mortality (%)	36.2	18.2	17.9	17.6	
		*p* = 0.032 (q1 vs q2)	*p* = 0.028 (q1 vs q3)	*P* = 0.030 (q1 vs q4)	

*Hb * hemoglobin, *q* quartile, *SD* standard deviation, *BMI* Body Mass Index, *BNP* B-type natriuretic peptide, *eGFR* estimated glomerular filtration rate, *MNA-SF* Short-form Mini-nutritional assessment, *MMSE* Mini Mental State Examination

## Discussion

We found that anemia is common in people living in nursing homes in Sweden. The overall anemia prevalence in this study was about the same, or lower, especially among women, compared to other earlier studies in nursing homes [17–23].

In our sample anemia was more common in men than in women. This difference is explained by the higher anemia limit for men defined by WHO [8]. It has been shown previously [14] that a higher overall prevalence of anemia in older men results from the sex-specific cut-points used to define anemia, with hemoglobin levels of 120 to 130 g/L (12–13 g/dL) defined as anemia in men but normal in women. Even minor changes in anemia criteria would result in significant increase or decrease in the prevalence of anemia.

We found a higher mortality rate among men with anemia compared to non-anemic men. This difference was significant even when adjusting for the covariates age, increased BNP and decreased eGFR. We did not see the same higher mortality rate among women with anemia.

We found no other studies describing sex differences regarding anemia as a risk factor for mortality in a nursing home population. Some other authors have shown similar sex differences in Hb to those in this study, both a higher anemia rate in elderly men than in women [6, 10, 14, 16, 34] using the WHO anemia limits, and a slightly higher mortality risk in anemic men than in women [16]. However, three of these studies were published in 1987–1999 and none of these studies was performed in a nursing home population. A Japanese case–control study performed in 1990–1996 on nursing home anemia subjects showed similar survival curves during a follow-up period for death rates of 5 years. The study was smaller, based on a lower anemia limit (110 g/L) for both sexes, involved few men and did not describe the sex differences [35].

In our study increased BNP or decreased eGFR were more common among men with anemia, but there was a higher mortality even after adjustment for these variables. This indicates that men in nursing homes with anemia may have more severe underlying causes of anemia than women. This is consistent with previous conclusions that the higher frequency of anemia in men can be explained by a higher prevalence of underlying diseases [16]. However, we cannot rule out the possibility that our results regarding gender differences and anemia versus mortality could be explained by that the gender specific cut-off levels for anemia is not relevant in this population. Furthermore, the observational design of this study precludes any definite conclusions about causality.

We also found that Hb decline during the last year was correlated with a doubled one-year mortality. Interestingly a higher Hb value was more associated with Hb decline than were low Hb values. These results are consistent with a previous study in a community-dwelling elderly but younger population (mean age 72.1 years) [36]. This means that it is difficult to predict declining Hb based on the current Hb level. Neither did we find other risk factors for Hb decline.

A low Hb value in an older person living in nursing home is a marker of disease and a warning signal for death, especially among men. A higher Hb value is no protection against a drop in Hb, which also is a separate warning signal and correlated with higher mortality. There may be a risk that low Hb values or Hb decline is overlooked and not further investigated in a nursing home population. The symptoms of anemia are vague in elderly patients and the onset of symptoms is usually insidious [11]. Other contributing factors may be complex comorbidity, a belief that the investigation results do not change treatment options and lack of time.

This study has some limitations. First, this is a rather small sample of nursing home residents especially when separating men and women in the analysis. Second, there may be a selection bias initially because palliative patients were excluded. The reason for moving into a nursing home may differ between men and women. Somatic disease may theoretically be a more common reason for nursing home care among men, while psychosocial factors such as loneliness and need for stimulation may matter more to women as they live longer. The lower anemia rate among the women in this study compared to other nursing home studies may support this hypothesis.

Third, the sample of nursing homes used was not randomly selected, but rather selected for reasons of convenience from three different areas in Sweden, with persons in nursing homes whose staff were interested in joining the project being asked to participate. However, the nursing homes included did not differ notably from other nursing homes in Sweden [28].

Since anemia is common among older adults, especially in nursing homes, further research is needed in this area. More knowledge about causes of anemia in a nursing home population would be valuable. More research is also required to establish guidelines on how to handle anemia in a multimorbid nursing home population.

## Conclusions

Our study suggests that anemia is common in nursing home residents in Sweden, as shown in previous studies in other countries. In our sample anemia was more frequent among men for whom it was related to a higher mortality.

A rapid Hb decline was associated with higher mortality. Hb decline was associated with higher Hb at baseline. According to our results it seems important to monitor Hb regularly in a nursing home population regardless of earlier Hb values.

## Abbreviations

ASA: Acetylsalicylic acid
BMI: Body mass index
BNP: B-type natriuretic protein
CKD: Chronic kidney disease
CRP: C-reactive protein
eGFR: Estimated glomerular filtration rate
GFR: Glomerular filtration rate
Hb: Hemoglobin
HSE: Health Survey for England
MMSE: Mini Mental State Examination
MNA-SF: Short-form Mini-nutritional assessment
NHANES III: The Third National Health and Nutrition Examination Survey
NSAID: Nonsteroidal anti-inflammatory drugs
SD: Standard deviation
SHADES: Study of Health and Drugs in the Elderly
SSRI: Selective serotonin re-uptake inhibitors
WHO: World Health Organization
